# The Effects of Light Therapy on Cognitive Function and Stress in Women With Breast Cancer Before Systemic Treatment

**DOI:** 10.1002/cam4.71412

**Published:** 2025-11-23

**Authors:** Snaefridur Gudmundsdottir Aspelund, Thorhildur Halldorsdottir, Gudjon Agustsson, Hannah Ros Sigurdardottir Tobin, Lisa M. Wu, Ali Amidi, Kamilla R. Johannsdottir, Susan K. Lutgendorf, Rachel Telles, Huldis Franksdottir Daly, Kristin Sigurdardottir, Mariana G. Figueiro, William H. Redd, Heiddis B. Valdimarsdottir, Birna Baldursdottir

**Affiliations:** ^1^ Department of Psychology, School of Social Sciences Reykjavik University Reykjavik Iceland; ^2^ Department of Oncology Aarhus University Hospital Aarhus Denmark; ^3^ Sleep & Circadian Psychology Research Group, Department of Psychology and Behavioural Sciences Aarhus University Aarhus Denmark; ^4^ Department of Psychological and Brain Sciences University of Iowa Iowa City Iowa USA; ^5^ Breast Center, University Hospital of Iceland Reykjavik Iceland; ^6^ Light and Health Research Center, Department of Population Health Science and Policy Icahn School of Medicine New York USA; ^7^ Department of Population Health Science and Policy Icahn School of Medicine New York USA

**Keywords:** α‐amylase, cancer‐related cognitive impairment, circadian rhythms, cortisol, internalizing symptoms, light therapy, surgery

## Abstract

**Background:**

Cancer‐related cognitive impairment (CRCI), for example, impairments in reaction time, processing speed, memory and executive function, may be associated with breast cancer (BC) surgery which can disrupt biological and psychological stress markers. Evidence suggests that light therapy may ameliorate cognitive impairment and stress. In this double‐blind, randomized controlled trial, we investigated the efficacy of light therapy on mitigating the impact of BC surgery on CRCI in a national Icelandic cohort of women with BC.

**Methods:**

Participants were randomly allocated to receive circadian‐effective blue light (BL, *N* = 60) or circadian‐ineffective dim light (DL, *N* = 57) for 4 weeks after surgery. The primary outcome was overall cognitive performance (assessed via global composite score), and secondary outcomes were specific cognitive domains, cognitive complaints, psychological (depressive symptoms, overall cancer‐related stress and its symptoms: hyperarousal, avoidance, and intrusive thoughts) and biological (cortisol and α‐amylase) stress markers. Linear regression and path analyses within structural equation modeling frameworks were conducted, adjusted for baseline cognitive performance, age, education, subsequent cancer treatment, cancer stage, and treatment credibility.

**Results:**

No group differences were found in overall cognitive performance or in specific cognitive domains, except for a non‐significant trend for faster reaction times in the BL group (*p* < 0.11). Additionally, the BL group reported significantly fewer cognitive complaints compared with the DL group (*p* < 0.05), as well as a non‐significant trend for less intrusive thoughts (*p* < 0.11). No group differences were observed in the biological stress markers.

**Conclusion:**

Overall, these findings suggest that light therapy may help mitigate the adverse effects associated with BC surgery on CRCI and intrusive thoughts, although further research is warranted.

**Trial Registration:** NCT04418856

Cancer‐related cognitive impairment (CRCI), such as impairments in reaction time, processing speed, attention, working memory, verbal memory, and executive function [1], is a common and potentially long‐lasting side effect of breast cancer (BC) and its treatment [2, 3]. Although previous research has primarily focused on the effects of chemotherapy on CRCI [4], CRCI has also been observed among women with BC prior to any treatment [5, 6] and following BC surgery [7, 8]. For instance, one study found that post‐operative women with BC were three to four times more likely to experience cognitive impairment compared with a healthy comparison (HC) group [9]. Additionally, a brain imaging study revealed structural changes in the thalamus of post‐operative women with BC, which were linked to attentional dysfunction (compared with HC) [8].

Although CRCI may arise from a myriad of complex and interconnected factors, psychological and biological stress are modifiable risk factors that may contribute to CRCI [6, 10, 11, 12]. The experience of receiving a cancer diagnosis and undergoing treatment can be highly stressful and traumatic [13]. Consequently, depressive and cancer‐related stress (related to posttraumatic stress disorder [PTSD]) symptoms are common among women with BC [14, 15] and have been associated with CRCI [6, 16, 17]. Biologically, acute and prolonged stress from cancer and its treatment can disrupt the stress reactivity systems, specifically the hypothalamic–pituitary–adrenal (HPA) axis and the autonomic nervous system [18, 19, 20], as reflected by their biomarkers, cortisol and α‐amylase [21, 22]. Cortisol, a glucocorticoid hormone regulated by the HPA axis [21] and α‐amylase, a salivary enzyme [22, 23], have both been linked to cognitive performance in patients with cancer [6, 24]. Disruption in biological stress can affect (cortisol‐receptor‐dense) brain areas important for cognitive function, such as the prefrontal cortex and hippocampus. For instance, higher cortisol levels have been shown to independently predict poorer cognitive performance in post‐operative testicular patients with cancer [24], and elevated cortisol has been associated with worse cognitive performance in non‐cancerous populations [25, 26]. Although the role of cortisol in cognitive function is well established [27], the role of α‐amylase is less studied. Recent studies have found α‐amylase in the hippocampus, where it is involved in glycogen metabolism, a process critical for energy supply during memory formation [28]. In our baseline study using the same sample [6], a flatter diurnal α‐amylase slope was associated with poorer overall cognitive performance. Additionally, elevated α‐amylase levels have been linked to mild cognitive impairment [29] and decreased cognitive performance [30] in various populations.

Surgical procedures may further contribute to CRCI by inducing a systemic stress response with increased cortisol production [31], through anesthesia [32], or by exacerbating circadian rhythm (CR) disruptions, which are common among women with BC [33, 34]. These disruptions illustrate the bidirectional relationship between the stress response and circadian systems. Normally, the circadian system regulates the stress response, activating it before the day begins and reducing it at night, thus optimizing energy and adapting to daily cycles [35]. Both cortisol and α‐amylase follow specific circadian patterns, with cortisol peaking in the morning and gradually decreasing throughout the day [36], whereas α‐amylase shows the opposite pattern (with lower levels in the morning that gradually increase and peak in the afternoon) [23]. CR is generated in the suprachiasmatic nucleus (SCN) [37] and entrained to the environment via zeitgebers, the strongest being light [38]. When light enters the eye, it activates the non‐image forming photoreceptor system projecting to the SCN and other brain areas involved in alertness, influencing the sleep/wake cycle and cognitive function (see [39] for an overview).

Given that light is the strongest zeitgeber of the circadian system [38], light therapy emerges as a promising low‐burden treatment for CRCI. Light therapy has been shown to influence cognitive function among other participant groups [40, 41, 42]. Evidence suggests that light therapy can synchronize disrupted CR among institutionalized individuals with major neurocognitive disorder [43, 44] and prevent CR from desynchronizing during chemotherapy for BC [45]. Additionally, studies have indicated that circadian‐effective bright light, can prevent fatigue, sleep quality, and quality of life from worsening among women with BC during chemotherapy, compared with non‐circadian stimulating dim red light [46, 47, 48]. Regarding the potential of light therapy to ameliorate stress, light therapy has been found to regulate cortisol levels among healthy individuals [49, 50] and decrease PTSD symptoms among veterans, as well as augment cognitive behavioral therapy for individuals with PTSD and panic disorders [51, 52]. Moreover, one study found that environmental bright light reduced the severity of depressive symptoms among hospitalized patients with multiple myeloma compared with circadian‐ineffective dim light (DL) [53].

The current study is the first (that we know of) to examine whether circadian‐effective blue light (BL) can mitigate the impact of CRCI following BC surgery using a circadian‐ineffective DL comparison condition. The primary aim of the present double‐blind randomized controlled trial was to test the efficacy of a 4‐week light therapy intervention on CRCI (i.e., overall cognitive performance assessed via global composite score) among post‐operative women with BC. We hypothesized that circadian‐effective BL would mitigate the negative impact of BC (i.e., the stress associated with the diagnosis, the surgical procedure, and associated factors) on cognitive function, predicting better overall cognitive performance, reaction time, processing speed, working memory, verbal memory, and fewer cognitive complaints in women receiving BL [54, 55], compared with those receiving the circadian‐ineffective DL. Additionally, we expected that the BL would mitigate the negative effects of BC (i.e., the stress associated with the diagnosis, surgery, and associated factors) on biological and psychological stress markers, with the BL group exhibiting reduced biological (via steeper diurnal α‐amylase and cortisol slopes) and psychological stress (i.e., depressive symptoms, overall cancer‐related stress and its symptoms: hyperarousal, avoidance, and intrusion) compared with the DL group [49, 50, 51, 53].

## 1 Materials and Methods

### 1.1 Participants

**FIGURE 1:**
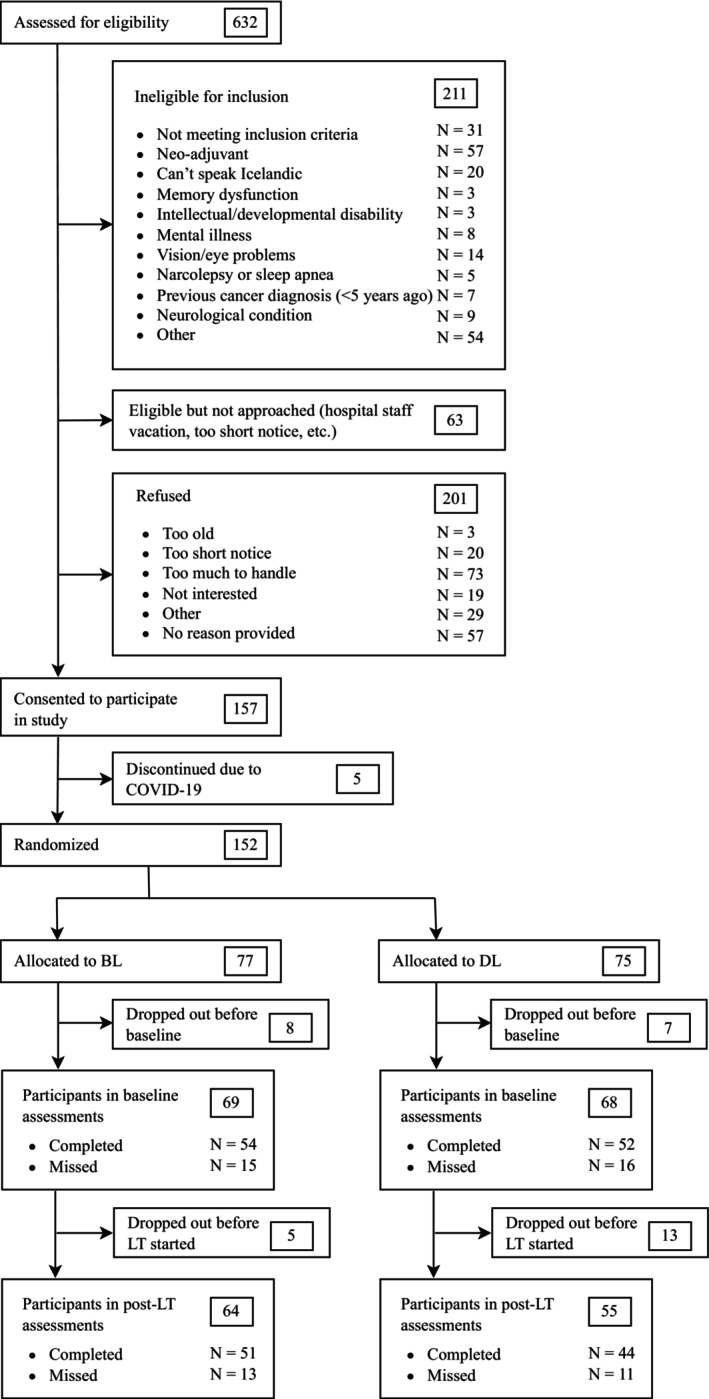
Participant flow through the study. BL, blue light; DL, dim light; LT, light therapy. The sample size consists of participants who completed either baseline or follow‐up (or both).

Eligible participants were newly diagnosed women with BC (Stages I–III) scheduled for surgery. All newly diagnosed women with BC in Iceland were invited to participate. Participants were excluded if they were scheduled to undergo a BC treatment other than surgery as their first treatment (i.e., neo‐adjuvant), if they had any contraindications for light therapy or a neurological condition that could influence neuropsychological functioning (e.g., autism diagnosis, epilepsy, and traumatic brain injury), age younger than 18, if they had other cancers or were undergoing treatment for another cancer, current pregnancy, pre‐existing anemia, shift work, eye diseases limiting the ability to process light, sleep disorders (e.g., narcolepsy), history of bipolar disorder/mania, travel to another time zone during the study, lack of access to a phone or a computer, and inability to understand or read Icelandic. All participants provided informed consent, which included information about the study examining the effects of different light intensities on the psychological side effects of BC and its treatment. See Figure 1 for the participant flow through the study and Table 1 for a more detailed sociodemographic overview of the participants. The study was approved by the chief medical officer at the National University Hospital, the National Bioethics Committee (VSN‐18‐199) of Iceland and the Icelandic Data Protection Authority.

**TABLE 1:** Comparison of the demographic and clinical characteristics of the post‐operative women with breast cancer in the blue light (BL) and dim light (DL) groups.

	BL (*N* = 60)	DL (*N* = 57)	*p*
Age in years (M, SD)	61.5 (11.7)	61.5 (10.2)	0.98
*Currently partnered, N (%)*			0.28
Yes	38 (63.3)	32 (56.1)	
No	12 (20.0)	17 (29.8)	
*Education level, N (%)*			0.68
Primary	9 (15.0)	7 (12.3)	
Secondary	17 (28.3)	14 (24.6)	
University	24 (40.0)	28 (49.1)	
BMI (M, SD)	27.5 (5.71)	28.5 (4.36)	0.35
*Physical activity, N (%)*			0.25
None	8 (13.3)	8 (14.0)	
Once a week	8 (13.3)	2 (3.5)	
Twice a week	6 (10.0)	9 (15.8)	
≥ 3 times a week	28 (46.7)	30 (52.6)	
*Menopause, yes %*	40 (66.7)	40 (70.2)	0.06
**Biological (M, SD)**			
Cortisol	4.84 (1.85)	4.94 (2.08)	0.76
α‐amylase	144.99 (92.08)	125.88 (82.12)	0.20
**Psychological (M, SD)**			
Depressive symptoms	12.0 (9.42)	9.46 (7.68)	0.13
Anxiety symptoms	4.48 (4.32)	3.85 (3.70)	0.41
Cancer‐related stress	25.2 (14.7)	25.2 (14.6)	0.99
**Clinical (*N*, %)**			
*Cancer stage*			0.23
I	33 (55.0)	27 (47.4)	
II	20 (33.3)	27 (47.4)	
III	7 (11.7)	3 (5.3)	
*Type of surgery*			0.42
Lumpectomy	39 (65.1)	33 (57.9)	
Mastectomy	17 (28.3)	21 (36.8)	
*Subsequent BC treatment*			0.69
No treatment	10 (16.7)	7 (12.3)	
Endocrine	31 (51.7)	28 (49.1)	
Radio/chemotherapy	19 (31.7)	22 (38.6)	
HER‐2‐positive	5 (8.3)	6 (10.5)	0.76
Estrogen‐positive	54 (90.0)	52 (91.2)	0.72
Progesterone‐positive	44 (73.3)	42 (73.7)	0.83
**Light Exposure (M, SD)**			
Light glasses use (days)	21.0 (8.87)	15.7 (6.85)	0.29
Light glasses use (min.)	631 (266)	472 (205)	0.29
**Patient perception of LT (M, SD)**			
Treatment credibility (out of 10)	7.76 (1.45)	6.76 (2.21)	0.010*
Treatment satisfaction (out of 5)	2.0 (1.00)	2.0 (1.41)	0.10

Abbreviations: BL, blue light; BMI, body mass index; DL, dim light; HER‐2, human epidermal growth factor receptor 2; LT, light therapy; M, mean; SD, standard deviation.

* *p* < 0.05 (two‐sided).

### 1.2 Randomization and Blinding

The participants were randomly allocated to receive either BL or DL using a block randomization design. The research team members who administered the neuropsychological assessments were blinded to the group allocation of the participants.

### 1.3 Light Therapy

Light therapy was delivered via AYO glasses which safely emit light from LEDs 15 mm from the eye [56]. The circadian‐effective light glasses used by the experimental group (BL) emit narrowband light (peak *λ* = 470 nm) blue light with an irradiance of 250 μW/cm^2^ (120 lx). The circadian system is maximally sensitive to short‐wavelength light peaking at 460 nm; therefore, the light level needed to have an effect on the circadian clock is much lower than white light sources, typically found in the built environment [56, 57]. In contrast, the circadian‐ineffective sham glasses (DL) emit blue light with an irradiance of 2.5 μW/cm^2^ (1.16 lx), which is below activation of the circadian clock. Participants were asked to wear the glasses for 30 min per day, 7 days per week for 4 weeks after surgery.

### 1.4 Neuropsychological Assessments

The neuropsychological tests were chosen based on recommendations from the International Cancer and Cognition Task Force [58] and available translations. Sustained attention and reaction time were measured with the 5‐min Psychomotor Vigilance Task [59], processing speed with the computerized Trail Making Test‐A (TMT‐A) [60, 61], working memory with the Wechsler Adult Intelligence Scale‐Fourth Edition Digit Span subtest [62] and verbal memory with the Rey Auditory Verbal Learning Fluency Test [63].

### 1.5 Psychological Assessments

Cognitive complaints were assessed via Patient‐Reported Outcomes Measurement Information System (PROMIS)—Cognitive Function 8a [64]. T‐scores for cognitive complaints were calculated using Icelandic norms (*from the COVIDMENT project*) where higher scores indicated better self‐reported cognitive function. Depressive symptoms were measured with the Center for Epidemiological Studies Depression Scale (CES‐D) [65] and overall cancer‐related stress with the Impact of Events Scale‐Revised (IES‐R) [66] total score. As light therapy could potentially influence the cancer‐related stress symptoms differently, we conducted three separate path analyses for each of the three IES‐R subscales: intrusive thoughts, hyperarousal, and avoidance. Treatment credibility was measured at baseline with the first item of the Credibility/Expectancy Questionnaire (CEQ) [67] (i.e., “*How logical does the* light therapy *offered to you seem*”). The item “*How do you rate this treatment overall*?” from the Functional Assessment of Chronic Illness Therapy‐Treatment Satisfaction‐General (FACIT‐TS‐G) was utilized to assess treatment satisfaction [68]. Anxiety symptoms (included in Table 1 and when comparing completers with non‐completers in Supporting Information S1) were assessed with the Generalized Anxiety Disorder‐7 (GAD‐7) [69]. Unless otherwise specified, higher scores indicated more severe symptoms or traits. The internal consistency (Cronbach's *α*) of the psychological assessments ranged from 0.88 (CES‐D) to 0.96 (PROMIS‐Cognitive Function).

### 1.6 Cortisol and α‐Amylase

See our previous baseline study [6] for a description of the saliva sampling and processing. The women with BC were instructed to refrigerate the saliva samples and bring them to their next scheduled hospital appointment, if applicable. A research team member retrieved the saliva samples either from the hospital, the home of the participants or from the post office if participants lived outside the greater capital area.

### 1.7 Covariates

#### 1.7.1 Sociodemographic and Clinical Variables

Participants provided information regarding their sociodemographic background, including gender, age, education, relationship status, body mass index (BMI), physical activity, and menopause status. Age and education, commonly accounted for when testing cognitive function [70], were examined for their association with overall cognitive performance, reaction time, processing speed, working memory, and verbal memory. Additionally, we adjusted for age in the biological models as cortisol and α‐amylase responses can differ with age [71, 72].

#### 1.7.2 Clinical Variables

Clinical data regarding cancer stage, type of BC surgery, subsequent BC treatment, human epidermal growth factor receptor 2 (HER2), estrogen, and progesterone status were retrieved from the medical records of the participants. As BC stage (i.e., Stages I, II or III) and subsequent BC treatments (no further BC treatment (0), endocrine therapy (1) or chemotherapy/radiotherapy (2)) could potentially influence both cognitive function [73], as well as biological and psychological stress [74], we included these variables as covariates to adjust for their effects.

### 1.8 General Procedure

All assessments were conducted remotely due to COVID‐19 social restrictions and to limit participant burden. The self‐report assessment was conducted online via Research Electronic Data Capture (REDCap) [75] and the neuropsychological tests were conducted over the phone or on a computer (see [6] for more details). To minimize potential differences in chronotype, participants were allowed to choose their testing times (between 8:00 a.m. and 10:00 p.m.) for their participation in the neuropsychological assessment. Participants were instructed to select a time when they felt (or performed) their best, ideally between 10:00 a.m. and 04:00 p.m. The participants completed all baseline measurements before any BC treatment. The day after their BC surgery, participants were instructed to start light therapy while still in the hospital. The light therapy session using AYO glasses was administered through a light therapy smartphone application so the date and duration of the light sessions could be monitored. Participants were instructed to initiate the session as soon as possible after waking up, as light therapy is most effective shortly after awakening [76]. To ensure compliance, the smartphone application restricted the light therapy sessions to between 5:00 a.m. and 12:00 p.m. The light therapy session lasted for half an hour and participants were instructed to adhere to this treatment protocol daily for four consecutive weeks. After completing the 4‐week light therapy, participants were instructed to partake in the post‐light therapy assessments as soon as possible within 1 week.

### 1.9 Statistical Data Analysis

Descriptive statistics, independent samples *t*‐tests and Fisher's tests were used to compare the BL and DL groups on sociodemographic, clinical and psychological variables. See our previous publication on the same sample [6] for how the initial global composite score, diurnal cortisol and α‐amylase slopes were calculated. For the sake of consistency, all neuropsychological *z*‐scores were coded such that higher scores would reflect better performance. The post‐light therapy *z*‐scores were calculated as follows [54]:

$$Z-{\text{Score}}_{\text{Post}-\mathrm{LT}}=\left(\mathrm{Raw}\;{\text{score}}_{\text{Post}-\mathrm{LT}}-\mu_{\text{Baseline}}\right)/\sigma_{\text{Baseline}}$$

where μ represents the mean at baseline and σ the baseline standard deviation. A postlight therapy global composite score was calculated with the mean *z*‐scores of all neuropsychological test outcomes for participants with complete data. As the computerized neuropsychological tests were automated and undertaken remotely without supervision, time values exceeding 1.5 * interquartile range (IQR) from the mean (28 from PVT and 7 from the TMT‐A) were classified as outliers and excluded from the analyses. We corrected for multiple comparisons using the false discovery rate (FDR) with the Benjamini–Hochberg procedure.

In order to test the direct influence of treatment condition on post‐treatment function, while concurrently controlling for covariates, as well as handling missing data using maximum likelihood estimation (MLE) [77, 78], we utilized the *lavaan* R package to perform path analyses [79]. The Yeo–Johnson transformation was utilized on skewed data using the *caret* package in R [80], as it is particularly effective for variables consisting of both positive and negative values (i.e., diurnal slopes and *z*‐scores of the neuropsychological tests). As the responses to cognitive complaints and perceived treatment credibility were still skewed post transformation (most participants rated their perceived cognitive function and treatment credibility as high), they were categorized into binary variables (see Supporting Information S1 for details).

All statistical analyses were performed in R and conducted on an intention‐to‐treat (ITT) basis. Sensitivity analyses were conducted to test the robustness of the findings across different conditions, that is among women with BC with a minimum of 50% treatment adherence (*N* = 79) and among those who participated during the winter (*N* = 81), when daylight hours were under average globally, during October to March. Additionally, we conducted a complete case analysis (without MLE, to evaluate the impact of MLE handling the missing data) and lastly, compared the characteristics of completers to non‐completers (the only analysis not corrected for multiple testing).

First, analyses were conducted on autoregressive models containing only autoregressive paths from pre‐ to post‐light therapy for each outcome, and fit indices were examined. Second, the full model containing treatment condition and covariates was analyzed. To improve model fit, modification indices were examined to identify potential paths or relationships. Covariates were included based on theoretical rationale and their relationship with key outcomes. Third, fit indices between the autoregressive and full models were compared using likelihood ratio tests to determine the best‐fitting model. Model fit was assessed with standard indices for path analyses: a non‐significant *χ* ^2^, the Comparative Fit Index (CFI > 0.90), Tucker–Lewis Index (TLI < 0.95), root mean square error approximation (RMSEA < 0.05) and standardized root mean residual (SRMR < 0.08) [81].

The path analysis models evaluated cognitive performance—overall cognitive performance, reaction time, processing speed, working memory, and verbal memory—each in separate models. These models (including cognitive complaints) included the direct effects of treatment group (BL compared with DL) and baseline cognitive function on post‐treatment cognitive function, while controlling for age, education, BC stage, subsequent BC treatment and perceived treatment credibility. As the completers and non‐completers in overall cognitive performance and overall cancer‐related stress significantly differed in cognitive complaints, depressive and anxiety symptoms (see Table S5), these were additionally controlled for (i.e., cognitive complaints, depressive, and anxiety symptoms in the overall cognitive performance path analysis, and cognitive complaints in the overall cancer‐related stress path analysis). Additionally, the models allowed both age and BC stage to correlate with subsequent BC treatment as BC treatment is often dependent on the BC stage and age (age may influence BC treatment decisions through factors, such as physiological tolerance, comorbidities, and risk tolerance). Age was also allowed to correlate with education (e.g., considering younger women may be more educated than older women). The biological and psychological stress models followed the same structure, except education was not included, and age was only included in the biological stress models.

## 2 Results

### 2.1 Participant Characteristics

A total of 117 women with BC (age range: 25–81) participated before any BC treatment and again after a 4‐week light therapy following BC surgery. Participants were randomly allocated to receive either a circadian‐effective BL (*N* = 60) or circadian‐ineffective DL (*N* = 57). Missing data ranged from 6% for cognitive complaints to 52% for salivary cortisol. Table 1 summarizes the main participant characteristics and shows that groups did not differ on any sociodemographic, clinical, biological, or psychological variables, except for perceived treatment credibility, *p* = 0.010 (see Table 1). To adjust for this group difference in perceived treatment credibility, the variable was included in the path analyses.

### 2.2 Model Comparisons

The autoregressive models, which consist solely of autoregressive paths (how pre‐light therapy levels predict post‐light therapy levels), consistently showed perfect fit indices, indicating an excellent fit to the data. The full models, incorporating the treatment condition (BL vs. DL) and theoretically relevant covariates, also demonstrated excellent fit indices across most outcomes. However, in the overall cognitive performance model, the TLI = 0.90 and RMSEA = 0.06. Similarly, in the intrusive thoughts model, all fit indices were excellent, except for RMSEA = 0.05, indicating very good fits rather than excellent ones.

For a comparison between the autoregressive models and the full models, see Table S1 in Supporting Information S1. Likelihood ratio tests indicated no significant improvements in model fit (all *p* > 0.05) when the treatment condition and theoretically relevant covariates (i.e., age, education, BC stage, subsequent BC treatment, and perceived treatment credibility) were added, likely due to the already excellent fit of the autoregressive paths (as well as increased complexity). Efforts to simplify the model by removing variables (i.e., education, subsequent BC treatment, and BC stage) and their correlations, did not lead to a significantly better fitting model. Although the fit indices for the full models did not show significant improvements over the autoregressive models, we still interpreted the full models for two reasons. Firstly, because they included the treatment effects and secondly, as incorporating covariates is essential to adjust for confounding factors and ensure accurate estimates of the treatment effects on cognitive function and stress.

### 2.3 Path Analyses

To explore the efficacy of light therapy on cognitive function and stress among post‐operative women with BC, path analyses were conducted. These analyses tested the hypotheses that cognitive performance (i.e., overall cognitive performance, reaction time, processing speed, and working memory) would be better in the circadian‐effective BL compared with the circadian‐ineffective DL, that cognitive complaints would be reduced, and that psychological (i.e., depressive symptoms, overall cancer‐related stress, and its symptoms: hyperarousal, avoidance, and intrusion) and biological stress (via steeper diurnal α‐amylase and cortisol slopes) would also be lower.

Table 2 shows the differences between the BL and the DL groups in cognitive performance, cognitive complaints, as well as in psychological and biological stress after undergoing the 4‐week light therapy.

**TABLE 2:** Path analysis results highlighting group differences in cognitive performance, cognitive complaints, and biological and psychological stress markers between post‐operative women with breast cancer receiving blue light (BL, coded as 1) versus those receiving the dim light (DL, coded as 0).

	*β*	SE	*Z*	FDR *p*	*d*
*Cognitive*					
Overall cognitive performance	0.10	0.10	0.91	0.53	−0.03
Reaction time	0.24	0.19	2.02	0.08	0.49
Processing speed	0.15	0.19	1.44	0.24	0.06
Working memory	0.04	0.12	0.51	0.75	−0.04
Verbal memory	−0.04	0.17	−0.55	0.72	−0.28
Cognitive complaints	0.22	0.07	2.84	0.011*	0.23
*Biological stress*					
Diurnal cortisol slope	−0.05	0.12	−0.27	0.86	0.12
Diurnal α‐amylase slope	−0.21	2.49	−1.42	0.26	−0.29
*Psychological stress*					
Depressive symptoms	−0.11	0.21	−1.35	0.29	−0.11
Overall cancer‐related stress	−0.11	0.38	−1.50	0.22	−0.27
Intrusive thoughts	−0.16	0.05	−2.08	0.07	−0.36
Hyperarousal	−0.10	0.04	−1.21	0.35	−0.16
Avoidance	−0.12	0.06	−1.54	0.21	−0.27

Abbreviations: BL, blue light; DL, dim light.

* *p* < 0.05 (two‐sided).

Post‐light therapy, there were no significant group differences in overall cognitive performance, processing speed, working memory, or verbal memory (FDR *p* > 0.05). However, there were non‐significant trends in the BL group for faster reaction time (*β* = 0.24, SE = 0.19, *z* = 2.02, FDR *p* = 0.08), suggesting moderate effects (*d* = 0.49), when compared with the DL group. Furthermore, participants in the BL group reported significantly fewer cognitive complaints (*β* = 0.22, SE = 0.07, *z* = 2.79, FDR *p* = 0.011), reflecting a small effect (*d* = 0.23). In terms of psychological stress markers, the BL group exhibited a non‐significant trend for fewer intrusive thoughts (*β* = −0.16, SE = 0.05, *z* = −2.08, FDR *p* = 0.07) with small‐to‐moderate effects (*d* = −0.36). No significant group differences were observed in overall cancer‐related stress, other cancer‐related stress symptoms (i.e., hyperarousal and avoidance), or depressive symptoms. Lastly, there were no significant group differences in biological stress markers (i.e., diurnal α‐amylase and cortisol slopes) between the groups (FDR *p* > 0.05). Path analysis results from sensitivity analyses, as well as the characteristics of the completers compared with non‐completers are provided in Supporting Information S1.

## 3 Discussion

This is the first study to examine the efficacy of light therapy for CRCI among post‐operative women with BC in a double‐blinded randomized controlled trial. The hypothesis that circadian‐effective BL could mitigate the negative impact of BC surgery on cognitive performance compared with circadian‐ineffective DL was partially supported. Although no group differences were observed in overall cognitive performance, processing speed, working memory, or verbal memory, the BL group did demonstrate a non‐significant trend for faster reaction times, compared with the DL group. This aligns with prior studies suggesting that circadian‐effective (i.e., bright) light is associated with faster reaction times in the general population [55, 82, 83]. Additionally, the BL group also reported significantly fewer cognitive complaints (i.e., rated their own cognitive function better) compared with the DL group. Potential reasons why our study showed group differences (in reaction time and cognitive complaints), whereas the study of Wu et al. [54] found none could include (a) our larger sample, and (b) our use of DL instead of dim red light. As the authors themselves argued, long wavelength red light, such as employed in their study, may have enhanced the impact of subsequent short wavelength light (e.g., daylight) on cognitive function [54, 84] (although this hypothesis needs further investigation).

The hypothesis that BL could mitigate the negative effects of BC surgery by reducing biological stress (via steeper diurnal cortisol and α‐amylase slopes) and psychological stress (i.e., depressive symptoms and cancer‐related stress) compared with DL was partially supported. The BL group demonstrated a non‐significant trend for fewer intrusive thoughts (a symptom of cancer‐related stress) compared with the DL group (including the results from the sensitivity analyses provided in Tables S2 and S4). This finding warrants further research as intrusive thoughts may be a risk factor for poorer patient outcomes and worse quality of life in women with BC [85]. The fact that previous evidence has shown that circadian‐effective (i.e., bright) light can decrease PTSD symptoms among other participant populations [51, 52, 86] underscores the need to study this further. However, in the current study, no significant group differences were found in overall cancer‐related stress or other cancer‐related stress symptoms, that is, avoidance or hyperarousal.

In line with previous results from another cancer patient group (Non‐Hodgkin Lymphoma survivors), we found no group differences in depressive symptoms [87]. This contrasts with Valdimarsdottir et al. [53] who reported that environmental bright light (in comparison with DL) alleviated depressive symptoms in hospitalized patients with multiple myeloma. These differences could largely be attributed to the more controlled hospital environment of their study, which may also have facilitated better treatment adherence due to the use of environmental light. The controlled hospital setting might have minimized confounding variables, such as exposure to natural daylight, which could have varied widely for our participants.

Regarding biological stress, the finding of comparable cortisol steepness in the BL and DL groups is consistent with previous results among patients with cancer [88]. Although a sensitivity analysis among women with BC with at least 50% treatment adherence showed that the BL group demonstrated flatter diurnal α‐amylase slopes relative to the DL group, this was neither observed in the main analysis or other sensitivity analyses, and therefore requires further investigation. Although previous research did not find effects of light on α‐amylase either [89, 90, 91], these are not directly comparable to ours as they examined acute effects of light on α‐amylase, unlike our 4‐week light therapy intervention. Long‐term light exposure, like in our study, may help shift or strengthen the CR robustness underlying the α‐amylase secretion [23].

The strengths of the current study include its broad scope of the efficacy of light therapy on cognitive function and stress among women with BC, consisting of both self‐reported and objective cognitive function, as well as both biological and psychological stress markers. The study design is another merit, as it involves a double‐blinded randomized controlled trial with a population‐based sample of women with BC (where all newly diagnosed women with BC in Iceland were invited to participate), featuring a novel comparison condition, and utilizing an ITT analysis.

Limitations include that only 79 participants (67.5%, out of *N* = 117) adhered to a minimum of 50% of the light therapy treatment protocol, which might have influenced the findings. The study took place during the COVID‐19 pandemic, which likely impacted treatment adherence by introducing significant psychological and logistical challenges for these newly diagnosed women with BC. Recruiting participants prior to BC surgery is particularly difficult due to the urgency of starting treatment and the heightened anxiety associated with both the diagnosis and treatment [92]. Due to this small sample size (especially in the saliva sampling), the findings should be interpreted with caution. However, despite challenges with missing data and treatment adherence, it is noteworthy that the main findings generally paralleled the sensitivity analyses, reinforcing the reliability of our findings despite these limitations. Additionally, although differences in chronotype, which may influence cognitive function [93], were not addressed, participants were allowed to select their cognitive assessment times to minimize potential chronotype‐related variations. Another limitation of the current study was that, due to small sample size, the mechanisms underlying the effects of BL on cognitive function and intrusive thoughts could not be examined. BL may influence cognitive function and intrusive thoughts through several potential mechanisms. For example, BL could synchronize CR, modulate stress systems, and/or improve sleep quality [94]. However, as changes in these domains would likely also affect depressive symptoms, an alternative explanation is that BL enhanced attentional control through increased alertness [95], potentially reducing intrusive thoughts (via interference inhibition) [96]. This latter mechanism may explain why BL was associated with faster reaction times, reduced cognitive complaints, and fewer intrusive thoughts, without affecting depressive symptoms.

Future studies with larger sample sizes are needed to confirm our findings and, if replicated, to explore the underlying mechanisms. These future studies should assess chronotype and be adequately powered to comprehensively explore the impact of light therapy on cognitive function, as well as biological and psychological stress markers. Additionally, extending this investigation by incorporating inflammatory markers would be beneficial. Although the current study examined CRCI post‐surgery, it is important to recognize that more severe cognitive impairments might occur later in the cancer treatment trajectory, such as during chemotherapy. Therefore, future studies (incorporating a DL comparison group) could investigate whether the effects of light therapy are more pronounced during these later stages, where the potential for observable improvement may be greater. Moreover, to enhance the practicality and applicability of light therapy further, future research should also focus on understanding the obstacles to treatment adherence and developing motivational incentives to boost adherence.

In conclusion, the findings in this study suggest that light therapy holds promise for mitigating the effects of BC (including the stress associated with the diagnosis, surgery and associated factors) on cognitive function (i.e., cognitive complaints and reaction time), as well as on intrusive thoughts. These findings emphasize the importance of early intervention in the cancer treatment trajectory, as that might result in better long‐term patient outcomes. The observation that simply wearing light therapy glasses daily for half an hour for 4 weeks may lead to, albeit small to modest, improvements in mood and cognition among women with BC is promising. This potential benefit, coupled with the non‐invasiveness, cost‐effectiveness, and minimal side effects of light therapy, warrants further investigation into its clinical and practical implications among patients with cancer.

## Author Contributions

**Snaefridur Gudmundsdottir Aspelund:** conceptualization, investigation, funding acquisition, writing – original draft, writing – review and editing, visualization, validation, methodology, software, formal analysis, data curation, project administration. **Thorhildur Halldorsdottir:** conceptualization, investigation, funding acquisition, methodology, validation, visualization, formal analysis, software, project administration, writing – review and editing, data curation. **Gudjon Agustsson:** investigation, data curation. **Hannah Ros Sigurdardottir Tobin:** data curation, formal analysis, methodology, validation, visualization, investigation, writing – review and editing, software, project administration, funding acquisition. **Lisa M. Wu:** conceptualization, methodology, validation, visualization, writing – review and editing, formal analysis. **Ali Amidi:** conceptualization, methodology, validation, visualization, writing – review and editing. **Kamilla R. Johannsdottir:** conceptualization, methodology, validation, writing – review and editing. **Susan K. Lutgendorf:** methodology, validation, writing – review and editing, conceptualization. **Rachel Telles:** methodology, conceptualization, validation. **Huldis Franksdottir Daly:** conceptualization, investigation, methodology, data curation. **Kristin Sigurdardottir:** investigation, conceptualization, methodology. **Mariana G. Figueiro:** methodology, validation, writing – review and editing, conceptualization. **William H. Redd:** conceptualization, methodology, validation. **Heiddis B. Valdimarsdottir:** conceptualization, investigation, funding acquisition, methodology, validation, visualization, writing – review and editing, formal analysis, project administration, data curation, supervision, resources. **Birna Baldursdottir:** data curation, supervision, resources, conceptualization, investigation, funding acquisition, methodology, validation, visualization, writing – review and editing, formal analysis, project administration.

## Funding

This work was funded by the Icelandic Research Fund (grant number 184999‐051), the Icelandic Cancer Society's Research Fund, and the Reykjavik University Research Fund.

## Ethics Statement

The study conformed to the ethical standards of the Declaration of Helsinki and was approved by the chief medical officer at the National University Hospital, the National Bioethics Committee (VSN‐18‐199) of Iceland and the Icelandic Data Protection Authority.

## Consent

All participants provided informed consent.

## Conflicts of Interest

The authors declare no conflicts of interest.

## Supporting information


**Table S1:** Fit indices for autoregressive and full models across all outcomes.
**Table S2:** Path analysis results for the treatment groups, the experimental group who received circadian‐effective BL compared to the comparison group who received the circadian‐ineffective DL, *including only participants with a minimum of 50% treatment adherence*.
**Table S3:** Path analysis results for the treatment groups, the experimental group who received circadian‐effective BL compared to the comparison group who received the circadian‐ineffective DL, *on participants who participated during winter*.
**Table S4:** Path analysis results for the treatment groups, the experimental group who received circadian‐effective BL compared to the comparison group who received the circadian‐ineffective DL, *on complete data without maximum likelihood estimation to handle any missingness*.
**Table S5:** Comparison of characteristics between completers (participants who met the minimum 50% light therapy adherence threshold, or provided data for the Global composite score, saliva sampling, computerized cognitive performance, and overall cancer‐related stress measures) and non‐completers (participants who did not).

## Data Availability

Due to compliance with the Icelandic data protection laws and the approval conditions set by the National Bioethics Committee of Iceland for this study, the data cannot be publicly shared. However, deidentified data may be made available to researchers who submit a methodologically sound proposal. Notably, access to the data is contingent upon approval by the National Bioethics Committee of Iceland. Researchers interested in accessing the data should direct their proposals to heiddisb@ru.is.
