# Supplementary material for: The Effects of Light Therapy on Cognitive Function and Stress in Women With Breast Cancer Before Systemic Treatment
Source: Cancer Med. 2025 Nov 23;14(22):e71412. doi: 10.1002/cam4.71412 (PMC12640611; doi:10.1002/cam4.71412)
Supplement: Supplementary file 1 — Table S1: Fit indices for autoregressive and full models across all outcomes. Table S2: Path analysis results for the treatment groups, the experimental group who received circadian‐effective BL compared to the comparison group who received the circadian‐ineffective DL, including only participants with a minimum of 50% treatment adherence. Table S3: Path analysis results for the treatment groups, the experimental group who received circadian‐effective BL compared to the comparison group who received the circadian‐ineffective DL, on participants who participated during winter. Table S4: Path analysis results for the treatment groups, the experimental group who received circadian‐effective BL compared to the comparison group who received the circadian‐ineffective DL, on complete data without maximum likelihood estimation to handle any missingness. Table S5: Comparison of characteristics between completers (participants who met the minimum 50% light therapy adherence threshold, or provided data for the Global composite score, saliva sampling, computerized cognitive performance, and overall cancer‐related stress measures) and non‐completers (participants who did not). [file CAM4-14-e71412-s001.pdf]

Supporting Information S1

2.10 Statistical data analysis continued

Cohen's *d* effect sizes were interpreted as follows: 0.2 was considered small, 0.5 moderate, and 0.8 large (1). Since the responses to cognitive complaints were still skewed post transformation, they were categorized into two groups based on a clinical cut-off score (a score of ≥ 45 was categorized as being within normal limits vs. below) (2,3). Additionally, given that a significant proportion of participants rated their perceived treatment credibility higher than 7, it was also categorized into two groups (≥ 7 vs. others). Data were assumed to be missing at random. The dependent variables were treated as fixed effects in the path analysis models and continuous independent variables were mean-centered. To address non-normality and variance heterogeneity of the path analysis model results for cognitive complaints and cortisol, 1,000 bootstrap samples were employed to estimate the standard errors. Additionally, to enhance the robustness and reliability of the findings from these models, the maximum iterations for the expectation-maximization algorithm were set to 10,000. Min-max scaling was applied to age since its variance was notably higher than that of the rest of the variables in the cortisol model.

The minimum 50% treatment adherence threshold was defined as using the light glasses for at least 20 minutes per day (out of the prescribed 30 minutes) on at least 14 of the 28 prescribed days (4,5).

Model comparisons

Table S1. Fit indices for autoregressive and full models across all outcomes.

Outcome	Model	χ² (df)	CFI	TLI	RMSEA	SRMR	LRT	FDR *p*
Cognitive								
Overall cognitive performance	Autoregressive	0.00	1.00	1.00	0.00	0.00	-	-
	Full	19.45 (14)	0.95	0.90	0.06	0.05	19.45	1.00
Reaction Time	Autoregressive	0.00	1.00	1.00	0.00	0.00	-	-
	Full	6.58 (8)	1.00	1.05	0.00	0.04	6.58	1.00
Processing speed	Autoregressive	0.00	1.00	1.00	0.00	0.00	-	-
	Full	4.80 (8)	1.00	1.09	0.00	0.03	4.80	0.92
Working memory	Autoregressive	0.00	1.00	1.00	0.00	0.00	-	-
	Full	6.04 (8)	1.00	1.04	0.00	0.04	6.04	0.84
Verbal memory	Autoregressive	0.00	1.00	1.00	0.00	0.00	-	-
	Full	6.36 (8)	1.00	1.03	0.00	0.04	6.36	0.99
Cognitive complaints	Autoregressive	0.00	1.00	1.00	0.00	0.00	-	-
	Full	7.05 (8)	1.00	1.02	0.00	0.04	7.05	1.00
Biological stress								
Diurnal cortisol slope	Autoregressive	0.00	1.00	1.00	0.00	0.00	-	-
	Full	1.02 (3)	1.00	1.25	0.00	0.02	1.02	0.80
Diurnal α-amylase slope	Autoregressive	0.00	1.00	1.00	0.00	0.00	-	-
	Full	1.82 (3)	1.00	1.12	0.00	0.03	1.82	0.88
Psychological stress								
Depressive symptoms	Autoregressive	0.00	1.00	1.00	0.00	0.00	-	-
	Full	1.86 (3)	1.00	1.05	0.00	0.03	1.86	1.00
Overall cancer-related stress	Autoregressive	0.00	1.00	1.00	0.00	0.00	-	-
	Full	3.11 (4)	1.00	1.02	0.00	0.03	3.11	1.00
Intrusive thoughts	Autoregressive	0.00	1.00	1.00	0.00	0.00	-	-
	Full	3.93 (3)	0.99	0.97	0.05	0.04	3.93	1.00
Hyperarousal	Autoregressive	0.00	1.00	1.00	0.00	0.00	-	-
	Full	2.10 (3)	1.00	1.04	0.00	0.03	2.10	1.00
Avoidance	Autoregressive	0.00	1.00	1.00	0.00	0.00	-	-
	Full	3.03 (3)	1.00	1.00	0.01	0.03	3.03	1.00

CFI = Comparative Fit Index; TLI = Tucker-Lewis Index; RMSEA= Root Mean Square Error Approximation; SRMR = Standardized Root Mean Residual; LRT = Likelihood Ratio Test

Sensitivity path analyses

Table S2. Path analysis results for the treatment groups, the experimental group who received circadian-effective BL compared to the comparison group who received the circadian-ineffective DL, *including only participants with a minimum of 50% treatment adherence*.

	β	*SE*	*z*	*FDR p*	*d*
<u>*Cognitive*</u>					
Overall cognitive performance	0.08	0.12	0.60	0.72	-0.10
Reaction Time	0.22	0.21	1.72	0.15	0.38
Processing speed	0.18	0.22	1.39	0.27	0.23
Working memory	0.01	0.14	0.14	0.94	-0.09
Verbal memory	-0.07	0.18	-0.79	0.61	-0.26
Cognitive complaints	0.21	0.09	2.18	0.03*	0.24
<u>*Biological stress*</u>					
Diurnal cortisol slope	0.05	0.15	0.23	0.98	0.23
Diurnal α-amylase slope	−0.34	1.93	-2.33	0.04*	-0.64
<u>*Psychological stress*</u>					
Depressive symptoms	−0.09	0.24	-1.00	0.27	-0.20
Overall cancer-related stress	−0.14	0.43	-1.70	0.16	-0.34
Intrusive thoughts	−0.17	0.05	-2.03	0.07	-0.36
Hyperarousal	−0.13	0.04	-1.56	0.20	-0.27
Avoidance	−0.15	0.06	-1.69	0.16	-0.36

BL = Blue light; DL = Dim light
* = $p < 0.05$ (two-sided)

Table S3. Path analysis results for the treatment groups, the experimental group who received circadian-effective BL compared to the comparison group who received the circadian-ineffective DL, *on participants who participated during winter*.

	β	*SE*	*z*	*FDR p*	*d*
Cognitive<br>Overall cognitive function	0.01	0.14	0.05	0.97	0.09
Reaction Time	0.40	0.24	2.77	0.012*	0.62
Processing speed	0.10	0.20	0.92	0.51	-0.09
Working memory	0.02	0.15	0.02	0.99	-0.04
Verbal memory	-0.06	0.20	-0.62	0.69	-0.18
Cognitive complaints	0.24	0.08	2.51	0.02*	0.22
Biological stress<br>Diurnal cortisol slope	−0.26	0.14	-0.98	0.34	-0.26
Diurnal α-amylase slope	0.14	2.92	-0.80	0.58	-0.21
Psychological stress<br>Depressive symptoms	−0.07	0.25	-0.80	0.58	-0.11
Overall cancer-related stress	−0.05	0.43	-0.58	0.71	-0.22
Intrusive thoughts	−0.14	0.05	-1.55	0.21	-0.43
Hyperarousal	−0.02	0.05	-0.23	0.87	-0.11
Avoidance	−0.07	0.06	-0.85	0.55	-0.16

BL = Blue light; DL = Dim light
* = $p < 0.05$ (two-sided)

Table S4. Path analysis results for the treatment groups, the experimental group who received circadian-effective BL compared to the comparison group who received the circadian-ineffective DL, *on complete data without maximum likelihood estimation to handle any missingness*.

	β	*SE*	*z*	*FDR p*	*d*
Cognitive					
Overall cognitive function	-0.06	0.10	-0.51	0.76	0.03
Reaction Time	0.20	0.20	1.68	0.18	-0.49
Processing speed	0.16	0.19	1.55	0.23	-0.06
Working memory	0.07	0.13	0.87	0.56	0.04
Verbal memory	-0.01	0.17	-0.15	0.91	0.28
Cognitive complaints	0.25	0.08	2.88	0.006*	-0.23
Biological stress					
Diurnal cortisol slope	−0.12	0.12	-0.64	0.67	-0.12
Diurnal α-amylase slope	−0.17	2.60	-1.08	0.45	0.29
Psychological stress					
Depressive symptoms	−0.15	0.21	-1.72	0.17	0.11
Overall cancer-related stress	−0.12	0.44	-1.48	0.26	0.27
Intrusive thoughts	−0.20	0.05	-2.63	0.02*	0.36
Hyperarousal	−0.13	0.04	-1.57	0.22	0.16
Avoidance	−0.15	0.06	-1.87	0.12	0.27

BL = Blue light; DL = Dim light
* = $p < 0.05$ (two-sided)

Characteristics of completers compared to non-completers

Due to over 10% missing data in the GCS, computerized tests, saliva sampling, and overall cancer-related stress, we conducted analyses to determine if participants who completed these measures (completers) differed from those who did not (non-completers). In addition, we compared participants who met the minimum 50% treatment adherence threshold (completers) with those who did not (non-completers). To examine potential predictors of participation, we analyzed age, educational level, baseline cognitive performance (except for when testing participation in the GCS, since the GCS encompasses all cognitive performance outcomes), post light therapy cognitive complaints, depressive symptoms, cancer-related stress, and anxiety symptoms. To avoid potential practice effects, baseline cognitive performance was assessed instead of post light therapy measures. The GCS served as the default measure of cognitive performance, except when the computerized assessment (a part of the GCS) was the focus of analysis, then working memory served as a proxy measure. Welch two sample *t*-tests were conducted to compare the means of completers and non-completers on these measures.

Table S5 shows that completers and non-completers did not significantly differ regarding participation in the computerized cognitive performance, saliva sampling, or in treatment adherence ($p > .05$). However, when comparing the completers and non-completers in overall cognitive performance and overall cancer-related stress, they significantly differed in cognitive complaints, depressive, and anxiety symptoms.

Table S5. Comparison of characteristics between completers (participants who met the minimum 50% light therapy adherence threshold, or provided data for the Global composite score, saliva sampling, computerized cognitive performance, and overall cancer-related stress measures) and non-completers (participants who did not).

Measure	Participation outcome	Mean (completers)	Mean (non-completers)	*t*	*p*
Age	Min. 50% LT	60.40	63.50	-0.92	0.36
Educational level	Min. 50% LT	3.58	3.25	0.61	0.54
Cognitive complaints	Min. 50% LT	31.76	30.25	0.60	0.55
Anxiety symptoms	Min. 50% LT	2.74	4.45	-1.52	0.13
Depressive symptoms	Min. 50% LT	8.75	11.27	-0.96	0.34
Overall CRS	Min. 50% LT	19.58	21.72	-0.50	0.62
Baseline GCS (z-score)	Min. 50% LT	-0.00	-0.22	0.93	0.35
Age	GCS	60.10	64.30	2.50	0.01*
Educational level	GCS	3.76	3.07	-2.25	0.03*
Cognitive complaints	GCS	33.71	29.61	-2.51	0.02*
Anxiety symptoms	GCS	2.35	4.44	2.81	0.01*
Depressive symptoms	GCS	7.31	11.29	2.24	0.03*
Overall CRS	GCS	18.22	22.50	1.31	0.20
Age	Computerized testing	60.10	64.30	2.50	0.26
Educational level	Computerized testing	3.59	3.50	-0.14	0.89
Cognitive complaints	Computerized testing	33.21	26.50	-2.18	0.05
Anxiety symptoms	Computerized testing	2.65	5.60	1.82	0.10
Depressive symptoms	Computerized testing	7.76	15.50	2.05	0.07
Overall CRS	Computerized testing	18.97	26.11	2.01	0.07
Baseline working memory	Computerized testing	17.25	15.40	-1.73	0.11
Age	Saliva sampling	61.75	61.15	-0.37	0.71
Educational level	Saliva sampling	3.75	3.49	-0.92	0.36
Cognitive complaints	Saliva sampling	32.35	32.93	0.44	0.66
Anxiety symptoms	Saliva sampling	2.92	2.83	-0.16	0.87
Depressive symptoms	Saliva sampling	9.40	7.71	-1.24	0.22
Overall CRS	Saliva sampling	20.66	18.58	-0.75	0.46
Baseline GCS (z-score)	Saliva sampling	-0.03	0.02	0.40	0.69
Age	Overall CRS	61.18	61.61	0.28	0.78
Educational level	Overall CRS	3.61	3.54	-0.25	0.81
Cognitive complaints	Overall CRS	31.57	34.87	2.87	0.01*
Anxiety symptoms	Overall CRS	3.01	2.58	-0.76	0.45
Depressive symptoms	Overall CRS	8.86	7.43	-1.12	0.27
Baseline GCS (z-score)	Overall CRS	-0.06	0.07	1.24	0.22

GCS = Global composite score; CRS = Cancer-related stress; Min. = Minimum; LT = Light Therapy
* = $p < .05$ (two-sided)

References

1. Cohen J. Quantitative methods in psychology: A power primer. Psychological Bulletin. 1992;153–9.

2. Edelen MO, Harrison JM, Rodriguez A, Weir R, Lai JS, Langer MM, et al. Evaluation of PROMIS Cognitive Function Scores and Correlates in a Clinical Sample of Older Adults. Gerontol Geriatr Med. 2022 Aug 13;8:23337214221119057.

3. Rothrock NE, Cook KF, O'Connor M, Cella D, Smith AW, Yount SE. Establishing clinically-relevant terms and severity thresholds for Patient-Reported Outcomes Measurement Information System® (PROMIS®) measures of physical function, cognitive function, and sleep disturbance in people with cancer using standard setting. Qual Life Res. 2019 Dec 1;28(12):3355–62.

4. Ancoli-Israel S, Rissling M, Neikrug A, Trofimenko V, Natarajan L, Parker BA, et al. Light treatment prevents fatigue in women undergoing chemotherapy for breast cancer. Support Care Cancer. 2012 Jun 1;20(6):1211–9.

5. Wu LM, Valdimarsdottir HB, Amidi A, Reid KJ, Ancoli-Israel S, Bovbjerg K, et al. Examining the Efficacy of Bright Light Therapy on Cognitive Function in Hematopoietic Stem Cell Transplant Survivors. J Biol Rhythms. 2022 Oct;37(5):471–83.
